# Heritability of saccadic eye movements in spinocerebellar ataxia type 2: insights into an endophenotype marker

**DOI:** 10.1186/s40673-017-0078-2

**Published:** 2017-12-19

**Authors:** Roberto Rodríguez-Labrada, Yaimeé Vázquez-Mojena, Nalia Canales-Ochoa, Jacqueline Medrano-Montero, Luis Velázquez-Pérez

**Affiliations:** 1 Centre for the Research and Rehabilitation of Hereditary Ataxias, Calle Libertad 26, 80100 Holguín, Cuba; 2 School of Physical Culture, University of Holguín, 25th street 104, 80100 Holguín, Cuba; 3 Medical University of Holguín, Lenin Avenue 4, 80100 Holguín, Cuba

**Keywords:** Spinocerebellar ataxia type 2, Saccadic eye movements, Heritability, Familiarity, Endophenotype

## Abstract

**Background:**

Saccade slowing has been proposed as endophenotype marker in Spinocerebellar Ataxia type 2 (SCA2), nevertheless the heritability of this trait has not been properly demonstrated. Thus the present paper was aimed to assess the heritability of different saccadic parameters in SCA2.

**Methods:**

Forty-eight SCA2 patients, 25 preclinical carriers and 24 non-SCA2 mutation carriers underwent electronystagmographical assessments of saccadic eye movements as well as neurological examination and ataxia scoring. Estimates of heritability based on the intraclass correlation coefficients were calculated for saccade velocity, accuracy and latency as well as for age at disease onset from 36, 17 and 15 sibling pairs of SCA2 patients, preclinical carriers and controls, respectively.

**Results:**

Saccade velocity was significantly reduced in SCA2 patients and preclinical carriers, whereas decreased saccade accuracy and increased saccade latency were only observed in the patients cohort. Intraclass correlation coefficient for saccade velocity was highly significant in SCA2 patients, estimating a heritability around 94%, whereas for the age at ataxia onset this estimate was around 68%.

**Conclusions:**

Electronystagmographical measure of saccade velocity showed higher familial aggregation between SCA2 patients leading the suitability of this disease feature as endophenotype marker, with potential usefulness for the search of modifier genes and neurobiological underpinnings of the disease and as outcome measure in future neuroprotective clinical trials.

## Background

Endophenotypes are measurable components of a disease that have simpler associations to genetic underpinnings than the disease syndrome itself [1]. The term has been commonly applied in psychiatric genetics [2, 3] and recently in other diseases such as neurodegenerative disorders [4, 5].

Endophenotypes must to fit some criteria that define its validity and usefulness [1], such as: a) be associated with illness in the population, b) be heritable traits, c) be primarily state-independent (manifests in an individual whether or not illness is active), d) co-segregate within families, e) be found in non-affected family members at a higher rate than in the general population and f) be measured reliably. Moreover, considering that endophenotypes are biomarkers influenced by the same genetic factors conferring risk for a disease [1], its characterization offers several important advantages over clinical phenotypes in the understanding of gene pleiotropy, a well-established phenomenon of a single gene affecting multiple traits [6].

Among the hereditary ataxias, endophenotype term has been only applied in the Spinocerebellar Ataxia type 2 (SCA2) [7]. This autosomal dominant disease is caused by an unstable CAG repeat expansion on a coding region of *ATXN2* gene, with the consequent expression of abnormally large polyglutamine tract in the ataxin-2 protein. SCA2 patients present a progressive cerebellar syndrome, usually accompanied by a combination of other non-cerebellar features such as slow saccades, peripheral neuropathy and others [8, 9]. Several of these non-cerebellar clinical characteristics appear before the ataxia onset and define the prodromal stage of the disease [10, 11]. SCA2 reaches the worldwide highest prevalence in Holguín, Cuba, as result of a founder effect [12, 13], identifying the Cuban SCA2 population as a homogeneous resource for genetic studies.

Electronystagmographical assessments have led to characterize the saccade slowing as SCA2 endophenotype due to this trait affects almost all patients [7, 14] and is present in a high proportion of preclinical carriers [15]. In addition, saccade slowing exhibits a significant correlation with the CAG expansion size [7], and can be reliably measured by more than a decade before the ataxia onset [11]. Nevertheless, the heritability of saccade slowing in SCA2 has not been assessed, which limits its value as disease endophenotype. Thus, the aim of this study was to determine the heritability of saccadic abnormalities in SCA2 through the intraclass correlation method in a cohort of patients and preclinical carriers.

## Methods

### Participants

Forty-eight patients and 25 preclinical carriers were admitted to the Centre for Research and Rehabilitation of Hereditary Ataxias in Holguín (CIRAH) for this study. Twenty-four non-SCA2 mutation carriers from Holguin province were admitted as control group. The main demographical, clinical and molecular data of each group is shown in the Table 1. All procedures were in accordance with the declaration of Helsinki and the standards of the institutional Ethics Committee for Scientific Research from the CIRAH. All participants gave their written informed consent prior to the experiments.

**Table 1:** Main demographical, clinical and molecular characteristics of the enrolled subjects

Variable	SCA2 patients (*N* = 48)	Preclinical carriers (*N* = 25)	Healthy controls (*N* = 24)
Gender (female/male)	19/29	16/9	15/9
Age (years)	41.35 ± 9.79 (18–69)	36.88 ± 9.14 (24–61)	41.13 ± 11.05 (20–63)
Age at onset (years)	30.06 ± 9.06 (9–60)	NA	NA
Disease duration (years)	11.71 ± 6.08 (3–29)	NA	NA
SARA score	15.81 ± 5.59 (6–30)	0.76 ± 0.66 (0–2)	0
Unexpanded CAG repeat (units)	22.08 ± 0.92) (19–27)	22.84 ± 1.93 (22–29)	22.54 ± 1.76 (20–30)
Expanded CAG repeat (units)	39.81 ± 2.92 (35–47)	35.76 ± 1.90 (32–39)	NA

*For quantitative variables the mean* ± standard deviation and range (in parenthesis) are shown

*NA* Not applicable, *N* Number of subjects, *SARA* Scale for the assessment and rating of ataxia

### Clinical assessments

All subjects underwent standardized neurological examination [16] and the Scale for the Assessment and Rating of Ataxia (SARA) [17] to evaluate cerebellar signs.

### Electronystagmographical assessments

Horizontal saccades were recorded binocularly with a 2-channel electrooculography (EOG) device (Jaeger-Toennies, Germany), using Ag-AgCl electrodes over right and left outer canthus of the eyes. The EOG signal was amplified and bandpass filtered (0.2–70 Hz). The data were sampled at a frequency of 200 Hz with a time base of 1000 ms/division, sensitivity of 200 μV/division and a time constant of 8 s. To minimize head movements of participants during the recordings, a forehead and chin rest were used in all cases.

Subjects were asked to fixate the target in the central position and to redirect their gaze to the new location of the target as soon as it appeared in the periphery, and later back in the central position. Ten centrifugal saccades in both horizontal directions were registered at 60° predictable amplitudes of stimulus. EOG signals were calibrated for a horizontal angle of 30°. Each session of EOG lasted around only 6 min. Saccade latency, accuracy and maximal saccade velocity were analyzed.

### Heritability analysis

The heritability estimates were obtained by analysis of variance components of the saccade parameters and age at onset among sibships and the error variance within the sibships, allowing for the computation of the sibling intraclass correlation (ICC) [18]. ICC values above zero indicate that error variance within the sibships is lower than variation between unrelated sibships. Then, assuming that the contribution of shared environmental factors to within-sibship variability is negligible, doubling the value of the sibling ICC provides an estimate of the heritability, which upper bound is the familiarity [19]. As expanded CAG repeats have a significant influence on the saccade velocity [7, 14, 15], we used an standard linear regression model to adjust the effects of this genetic parameter on the saccade variables before the analyses.

Heritability analyses in SCA2 patients were conducted in 21 sibling groups, 17 of them had two members, three had three members and only one had five members, which yielded a total of 36 sibling pairs. Among preclinical carriers, a total of 17 sibling pairs were obtained from 11 sibling groups, eight of them with two members and three with three members. Finally, 15 sibling pairs of non-SCA2 mutation carriers were obtained from nine siblings groups with two members and two siblings groups with three members.

### Statistical analyses

Intergroup differences of frequencies for clinical features were computed by Chi-square (χ^2^) tests. One-way ANOVAs, followed by Fisher-LSD post hoc tests, were carried up to compare the saccade variables between groups. Correlation analyses (followed by Bonferroni correction) were performed by the Pearson correlation test. All analyses were conducted using the STATISTICA software package (StatSoft, Inc., 2003, v6).

## Results

### Clinical characterization

**Fig. 1:**
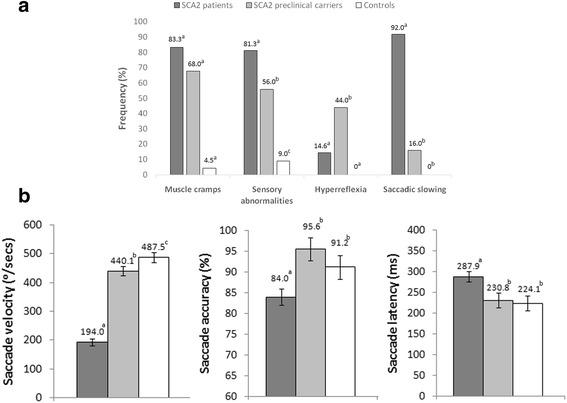
Intergroup comparisons of clinical (**a**) and electronystagmographical abnormalities (**b**). Bars represent the mean, and error bars the standard error of the means. The findings of X^2^ test and the Fisher LSD post-hoc comparisons are showed by letters (a,b,c) code. The same letter above distinct bars represents no significant differences (*p* > 0.05), whereas different letter means significant differences (*p* < 0.05)

The frequency comparisons of main clinical features between the groups are showed in the Fig. 1A. All SCA2 patients exhibited a cerebellar syndrome, characterized by gait ataxia, postural instability, dysarthria, dysmetria and dysdiadochocinesia. Consistent with previous studies [8, 9, 12], muscle cramps and sensory abnormalities were significantly reported by patients and preclinical carriers, as compared to controls. Hyperreflexia was only significantly detected in preclinical carriers, but not in patients due to the effect of the peripheral sensory neuropathy affecting more notably the patients than preclinical carriers [20]. It is known that SCA2 mutation carriers commonly show a conversion from deep tendon hyperreflexia to hyporeflexia even since late prodromal stage which is associated to the progression of sensory neuropathy [10]. Slowed horizontal saccades at bedside examination were highly frequent in SCA2 patients. Age at disease onset (*r* = 0.55; *p* < 0.0001) and SARA score (*r* = 0.42; *p* = 0.003) were significantly correlated to expanded CAG repeats in the patients’s cohort.

### Characterization of saccade abnormalities

Similar to preceding works [7, 14, 15], saccade velocity was significantly reduced in SCA2 patients and preclinical carriers, whereas decreased saccade accuracy and increased saccade latency were only observed in SCA2 patients (Fig. 1b).

Correlation analyses only disclosed significant associations of saccade velocities with expanded CAG repeats (*r* = 0.60; *p* = 0.0001), age at onset (*r* = −0.45; *p* = 0.002), aging (*r* = 0.59; p = 0.0001) and SARA score (*r* = −0.37; *p* = 0.012) in SCA2 patients.

### Heritability estimates.

Analyses of variance components of residual age at onset in SCA2 patients showed an ICC of 0.34, suggesting upper bounds of heritability or familiarity of ~68%. For saccade parameters, the data of heritability analyses are presented in Table 2.

**Table 2:** Heritability estimates of the saccade parameters in SCA2 patients, preclinical carriers and healthy controls

Variables	SCA2 patients			Preclinical carriers			Healthy controls		
	ICC	p	H	ICC	p	H	ICC	p	H
Saccade velocity (°/s)	0.47	0.002	~94%	0.19	0.222	*NA*	0.136	0.289	*NA*
Saccade accuracy (%)	0.16	0.170	*NA*	0.20	0.208	*NA*	0.013	0.473	*NA*
Saccade latency (ms)	0.16	0.170	*NA*	0.13	0.294	*NA*	0.286	0.121	*NA*

*ICC* Intraclass correlation coefficient, *H* heritability, *NA* Not attributable heritability estimates

The sibling ICCs were only significant for saccade velocity in the SCA2 patients, indicating that the within-siblings variability is significantly less than the between-siblings variability. Therefore, the upper bound of heritability for saccade velocity was ~94%. No significant ICCs were observed for the remaining saccade parameters in SCA2 patients as well as for all saccade parameters in preclinical carriers and controls.

## Discussion

This paper is the first study assessing the heritability of saccades in SCA2 or any hereditary ataxia. According to our results, 94% of the residual variance of saccade velocity is explained by genetic factors, regardless the expanded CAG repeats, and/or environmental factors shared by families, supporting the classification of this oculomotor trait as disease endophenotype.

Our findings contradict a previous paper [21] reporting significant differences in saccade velocity between two SCA2 monozygotic twin patients. This discrepancy could be explained by the reduced number of twin pairs enrolled and the existence of non-germline factors that differentially affect each twin, such as somatic mosaicism, epigenetic factors and differential heteroplasmy of disease-modifying mitochondrial mutations.

It is known that somatic mutations continuously occur along the life and twins may show different somatic mutation rates depending on environmental influence [22]. Indeed, previous evidences have suggested that somatic mosaicism of expanded CAG repeats is an important modifying factor of SCA2 disease progression [23–25] and its prevention by CAA-interruptions in the ATXN2 gene’s CAG tract seems to be related to the SCA2 parkinsonian phenotype [25].

Moreover, differential DNA methylation levels in the ataxin-2 gene promoter might also have modifying effects on the SCA2 phenotype [26–28]. For example, a significant disease onset anticipation was observed in one SCA2 family without intergenerational CAG repeat instability but distinct patterns of DNA-methylation in the ATNX2 gene [26]. Interestingly, epigenetic differences in discordant monozygotic twins have been reported for amyotrophic lateral sclerosis, a devastating late-onset neurodegenerative condition in which the ATXN2 gene can be also implicated, but not differential methylation within its specific promoter was observed among the discordant twins [29]. Likewise, epigenetic differences in monozygotic twins explain discordant phenotypes for Alzheimer disease [30], major depressive disorder [31] and schizophrenia [32]. Nevertheless, none of these works have the effects of the differential epigenetics factors in specific disease endophenotypes.

In contrast to the abovementioned work, a large study enrolling 112 normal twins showed significant heritability estimates for saccade velocity and other oculomotor traits [33]. Nevertheless, longitudinal genetic studies in twins are needed to assess more robustly the genetic factors underlying the individual variability of these parameters.

The lack of familial aggregation for saccade accuracy and latency in SCA2 patients could be explained by the higher heterogeneity of these measures, which are more susceptible to additional influences such as the visual feedbacks that modify the accuracy of slowed saccades at flight [34], as well as attentional levels [35] and rehabilitation effects [36] on the saccade latency.

The heritability of saccade velocity was higher compared with the age at onset’s estimates obtained in the present study and in previously studied cohort [37, 38]. These results suggest that saccade velocity may be more appropriate for genetic studies than age at onset in SCA2 and so, it’s use could result in more sensitive approaches to detect disease modifier genes and the neurobiological bases of SCA2.

The link between the genetic basis of SCA2 and the saccade slowing consists in the preferential vulnerability of saccadic excitatory burst neurons (EBN) to expanded ataxin-2 [39]. Within the saccadic circuitry, these neurons receive excitatory inputs from the caudal pole of the contralateral cerebellar fastigial nucleus, which is controlled by inhibitory inputs from the Purkinje cells and in turns, they drive abducens motoneurons to produce saccades [40]. Then, this susceptibility could be explained by the excitotoxic effects caused the deliberated cerebellar outputs as result of the loss of inhibitory control from the Purkinje cells [8, 9]. Additionally, expanded ataxin-2 could impair the calcium signaling pathways, mitochondrial function and bioenergetic sensoring in EBN cells [41–44], three key functions for these high-energy consuming neurons.

Therefore, the differences observed in the saccade velocity between SCA2 patients with relatively similar mutation sizes could result from differential effects of modifier genes on the PolyQ toxicity in the EBN cells. Hence, the study of patients who have saccade velocities out of the mean range adjusted on CAG repeats offers important advantages for the identification of these modifying genes and the understanding of its mechanisms of actions.

**Fig. 2:**
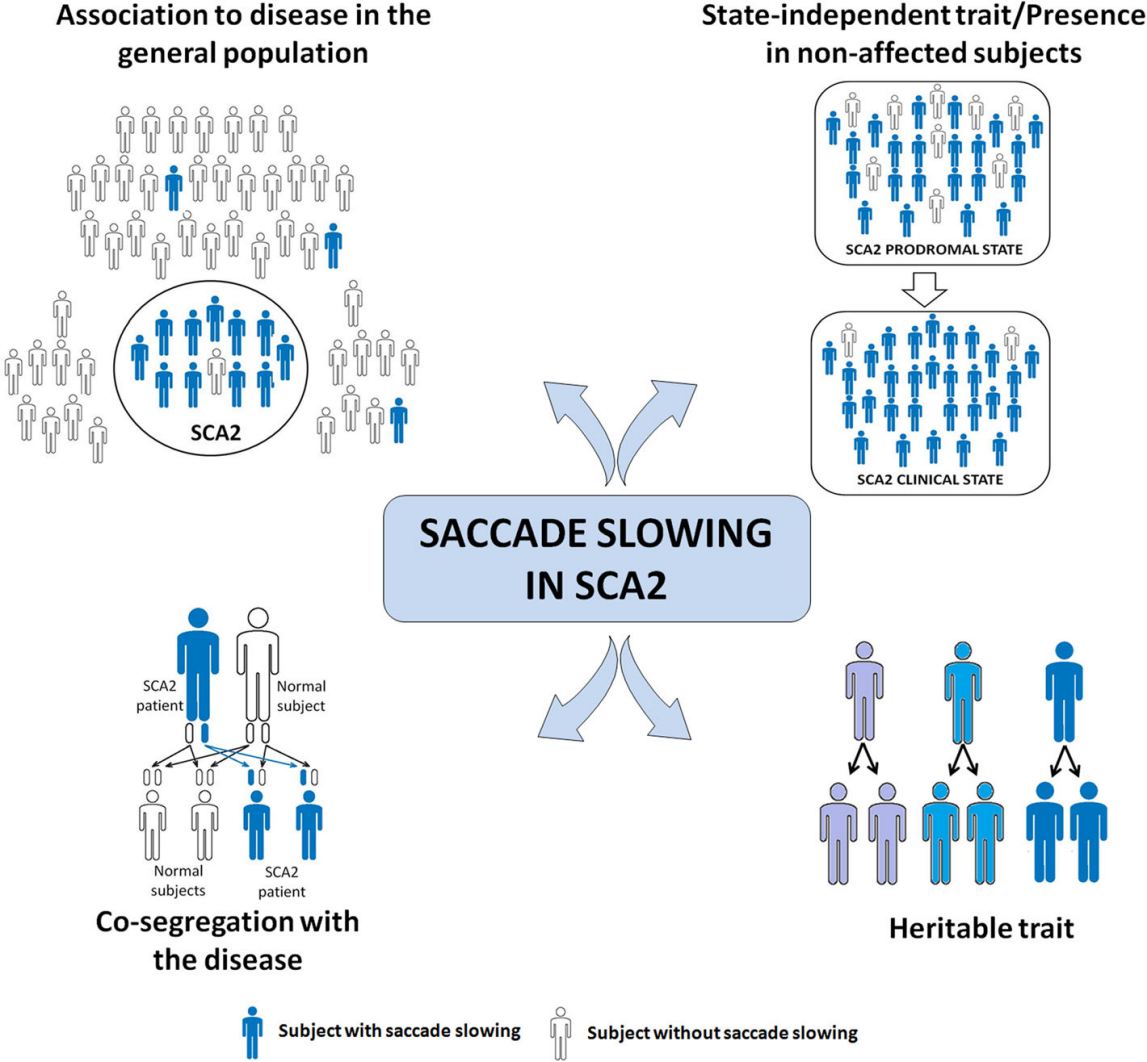
Criteria defining saccade slowing as endophenotypical marker in SCA2. White drawings denote subjects with normal saccade velocities, whereas blue drawings denote subjects with saccade slowing. Drawings with different blue shades denote different degrees of saccade slowing

Then, with the higher heritability estimates observed in this paper for saccade slowing, we satisfy all criteria defining endophenotypes, which were shown in Introduction and thus we offer new evidences about the conceptualization of this oculomotor trait as endophenotype marker in SCA2 (Fig. 2). As endophenotype, the saccade slowing confers neurobiological insights beyond the traditional symptomatic approaches of the disease and its assessment could be help to stratify SCA2 patients and preclinical carriers into more homogeneous cohorts, leading to more objective physiopathological approaches and meaningful clinical trials.

A limitation of this paper was the inclusion of a relative small number of subjects. Nevertheless, even in this small cohort, we obtained significant heritability estimates for saccade slowing, suggesting the robustness of this heritable measure.

## Conclusions

As conclusion, saccade slowing, as assessed by electronystagmography, showed higher familial aggregation between SCA2 patients, supporting the suitability of this oculomotor trait as disease endophenotype, a sensitive tool for the search of modifier genes and neurobiological underpinnings of the disease, as well as to evaluate the efficacy of future neuroprotective clinical trials.

## Abbreviations

CAG: Cytosine-adenine-guanine
EBN: Saccadic excitatory burst neurons
ICC: Intraclass correlation
PolyQ: Polyglutamine
SARA: Scale for the Assessment and Rating of Ataxia
SCA2: Spinocerebellar Ataxia type 2
